# A post-mortem survey on end-of-life decisions using a representative sample of death certificates in Flanders, Belgium: research protocol

**DOI:** 10.1186/1471-2458-8-299

**Published:** 2008-08-27

**Authors:** Kenneth Chambaere, Johan Bilsen, Joachim Cohen, Geert Pousset, Bregje Onwuteaka-Philipsen, Freddy Mortier, Luc Deliens

**Affiliations:** 1 End-of-Life Care Research Group, Vrije Universiteit Brussel, Belgium; 2 Bioethics Institute Ghent, Ghent University, Belgium; 3 VU University Medical Center, Department of Public and Occupational Health, EMGO Institute, Amsterdam, The Netherlands

## Abstract

**Background:**

Reliable studies of the incidence and characteristics of medical end-of-life decisions with a certain or possible life shortening effect (ELDs) are indispensable for an evidence-based medical and societal debate on this issue. This article presents the protocol drafted for the 2007 ELD Study in Flanders, Belgium, and outlines how the main aims and challenges of the study (i.e. making reliable incidence estimates of end-of-life decisions, even rare ones, and describing their characteristics; allowing comparability with past ELD studies; guaranteeing strict anonymity given the sensitive nature of the research topic; and attaining a sufficient response rate) are addressed in a post-mortem survey using a representative sample of death certificates.

**Study design:**

Reliable incidence estimates are achievable by using large at random samples of death certificates of deceased persons in Flanders (aged one year or older). This entails the cooperation of the appropriate administrative authorities. To further ensure the reliability of the estimates and descriptions, especially of less prevalent end-of-life decisions (e.g. euthanasia), a stratified sample is drawn. A questionnaire is sent out to the certifying physician of each death sampled. The questionnaire, tested thoroughly and avoiding emotionally charged terms is based largely on questions that have been validated in previous national and European ELD studies. Anonymity of both patient and physician is guaranteed through a rigorous procedure, involving a lawyer as intermediary between responding physicians and researchers. To increase response we follow the Total Design Method (TDM) with a maximum of three follow-up mailings. Also, a non-response survey is conducted to gain insight into the reasons for lack of response.

**Discussion:**

The protocol of the 2007 ELD Study in Flanders, Belgium, is appropriate for achieving the objectives of the study; as past studies in Belgium, the Netherlands, and other European countries have shown, strictly anonymous and thorough surveys among physicians using a large, stratified, and representative death certificate sample are most suitable in nationwide studies of incidence and characteristics of end-of-life decisions. There are however also some limitations to the study design.

## Background

The quality of medical care at the end of life has become of major importance in contemporary developed societies [1-5]. In the past century there has been a significant shift in cause of death, away from acute deaths due to infectious disease towards deaths caused by chronic and degenerative illness such as cancer and cardiovascular disease [1-3,6]. Combined with rising life expectancy and an ageing population, this epidemiological transition has resulted in an increased number of people experiencing a terminal illness phase at the end of life [1,6].

Parallel to these changes in the patterns of dying, advances in medical knowledge and technology have contributed considerably to the increase of treatment possibilities at the end of life. Physicians are now increasingly able to ensure effective treatment of pain and symptoms at the end of life, and to postpone a patient's death [1,2,4,6]. However, in many cases a point is reached where those involved feel that prolonging life is no longer desirable as a certain minimal quality of life cannot always be maintained [1,5-8]. This gives rise to decisions that possibly or certainly hasten the patient's death, i.e. end-of-life decisions (ELDs). These decisions include withholding or withdrawing potentially life-sustaining treatment, intensifying pain and/or symptom management with a possible life-shortening effect and administering drugs with the explicit intention of hastening death (i.e. physician-assisted suicide, life-ending without the patient's explicit request, and euthanasia).

Past studies in different countries have revealed that these various end-of-life decisions are made in a significant proportion of deaths [8-20], although incidence estimates vary somewhat across countries. According to the 2001 EURELD study in six European countries (Belgium, The Netherlands, Denmark, Italy, Sweden and Switzerland) the incidence of deaths preceded by an ELD ranges from 23% to 51% [14]. In Belgium the incidence rate dropped slightly, although not statistically significantly, from 39,3% to 38,4% between 1998 and 2001 [19]. These studies contributed to an ongoing ethical and legal debate concerning end-of-life decisions, culminating in Belgium in 2002 with the passing of the laws on palliative care, patients' rights and euthanasia (which permits euthanasia under strict conditions of prudent practice) [21-24].

It is in this new legal context that a third ELD study in Belgium was undertaken. This study is part of the larger **M**onitoring quality of **E**nd-of-**L**ife **C**are (MELC) study in Flanders [25], and aims to obtain reliable incidence estimates of ELDs and their characteristics in Flanders for 2007, as well as to take a closer look at the decision-making process preceding ELDs and the treatment and care provided at the end of life. As a third measurement point for Flanders, one of the research aims is to permit a trend analysis of end-of-life decision making. Furthermore, the legalisation of euthanasia since the last ELD study in Flanders creates the opportunity of estimating the possible effects of the euthanasia law on the practice of euthanasia and other end-of-life practices [24], and will shed light on the argument that legalising euthanasia will possibly lead to a slippery slope, e.g. a rise in life-ending acts without the patient's explicit request [26,27]. Comparison of the results of the Flemish study to the Dutch data from 2005 will put the findings in an international perspective [24].

To design an adequate methodology for a nationwide study of ELDs is not straightforward because of the sensitive nature of the issue and the specific difficulties involved in the organisation of such a survey. In this article we present the protocol of the 2007 Flemish ELD study, which was guided by four methodological questions: (1) which study design is most appropriate for obtaining reliable incidence estimates and descriptions of ELDs, even of rare ELDs, that are representative for all deaths in Flanders in 2007?; (2) how can comparability with earlier ELD studies in Flanders and other countries be ensured?; (3) how can strict anonymity of physicians and patients for ethical and judicial reasons be guaranteed?; and (4) how can a sufficient response rate for a survey on this sensitive subject be achieved?

The study design we present in this article is based on a method, first developed in the Netherlands in 1990 [9], that has been successfully used in several European countries to study the nationwide incidence and characteristics of ELDs [8-10,13-15,19,20]. However, this is the first time that this study design has been described in detail. We believe that presenting it will be useful to researchers in other countries who intend to embark on similar research. The methodology outlined in this article will also serve as a reference for future publications using data from this study.

## Method design

### A retrospective survey based on death certificates

Obtaining data from a representative sample of dying patients in a prospective study design is an impossible task, as this would entail following an excessively large number of patients in numerous care settings. Moreover, defining who is dying is never clear-cut, and the problems of patient burden and attrition or non-response of the sickest patients [28,29] rules out the option of a prospective study design. There is also a danger that a prospective study will influence the behaviour of physicians and other caregivers. A retrospective (post-mortem) study design was therefore the more favourable option for this study.

Because the study aims to obtain reliable estimates of ELDs for all deaths in Flanders, it was desirable to take the death case as the unit of measurement as this evidently provides a clear epidemiological denominator for the entire population of deaths, as well as for the subpopulations of deaths, e.g. cancer deaths. This provides more reliable incidence estimates than incidence studies where the physician is the unit of measurement and a representative sample of physicians are asked to report on the last death under their supervision in e.g. the last 12 months [11,12,18]. In these studies, the number of deaths per participating physician is often not taken into account, and ELD incidence rates on population level are estimated on the basis of physician characteristics. Also, recall bias can be considerable if the physician's last death occurred a long time before the study.

Every death in Belgium must be registered via a death certificate issued by the civil registrar of the municipality where the death took place. The physician completes the first part of the death certificate, indicating the sex of the deceased, some medical information (such as causes of death), time and place of death and signs the certificate with full name and medical registration number. The second part of the death certificate (containing socio-demographic information about the residence, age, education, occupation, nationality, civil status and living situation of the deceased) is completed by the civil registrar of the municipality in which the death took place. The death certificates are first processed by the provinces where the death occurred before they are sent to the central administration authorities. For Flemish death certificates this is the Flemish Agency for Care and Health (part of the Flemish Ministry for Welfare, Public Health and Family). Death certificates are thus particularly suitable for a nationwide study of ELDs; because every death is represented by a death certificate, it is easy to draw a representative sample of deaths. Also, the certifying physician's identification details listed on the death certificates allow the physician to function as the observational unit for the study. Furthermore, the socio-demographic and morbidity data of the deceased are readily available on the certificates and can be included in the survey. We obtained permission from the Flemish Agency for Care and Health to conduct a cross-sectional postal survey among the certifying physicians of a representative sample of death certificates.

### Selection of deaths and sampling

The selection of deaths and sampling procedure needed to provide a representative sample of all deaths in Flanders in 2007 and had to include a sufficient amount of deaths to yield reliable information on the characteristics of all types of ELDs. Inclusion criteria for the study were:

- the death taking place in Flanders,

- the deceased is a resident of Belgium at the time of death,

- the deceased is aged one year or more at the time of death.

The death must have occurred in Flanders as the aim of the study is to describe end-of-life practices in the Flemish region; the limited number of Flemish residents who died outside Flanders are thus not included. The criterion of residence in Belgium is necessary to exclude all deaths in Flanders of persons, with or without the Belgian nationality, who live abroad as their socio-demographic characteristics and medical history would not be available. The number of these deaths is very small anyway and the majority of them are caused by traffic accidents, indicating a low likelihood of an ELD preceding death. Deaths of neonates (under one year of age) are excluded because end-of-life decision making is a very different issue in this age group, requiring an adjusted questionnaire. ELD studies in neonates have been done in the past in Belgium and the Netherlands [24,30,31], but were not necessary for the present study.

We sampled a fraction of almost 25% in a six month period from June 1^st ^until November 30^th ^2007. This amounted to 6928 death cases, approximately 12% of all deaths in 2007 (percentages based on the mortality rate of Flemish deaths for 2006, the most recent reference year for which mortality statistics were available). The sample size and proportion are significantly larger than in the previous Flemish ELD studies [13,14,19], ensuring the greater overall statistical power of the results. The sample size necessary to estimate accurately the incidence rates with a confidence level of 95% was calculated based on the response level of the previous Flemish ELD studies in 1998 (49%) in 2001 (59%) [32].

The sample is proportioned for month of death and province of death (Flanders consists of five provinces). From the previous ELD studies we know that ELDs occur more frequently among patients with a certain cause of death [13,14]. We therefore adopted disproportionate sampling of deaths to include more patients with a cause of death known to have a higher likelihood of one or more ELDs. This should result in more cases in which an ELD preceded death, and should thus further increase the statistical power and reliability of the incidence estimates and descriptions, even for the less-prevalent ELDs. According to the underlying cause of death on the death certificates and the corresponding probability of an end-of-life decision being made (derived from the data of the Flemish 2001 ELD study) deaths are grouped into one of four strata and sampled disproportionately (see Table 1).

**Table 1:** Four strata for disproportionate stratification based on cause of death*

Stratum 0
Cause of death implies that an ELD is certain
Included causes of death: euthanasia**.
Every death in this stratum is selected for the survey.

Stratum 1
Cause of death implies that an ELD is probable
Included causes of death: neoplasms (ICD-10 codes: C, D00–D48).
One out of every two deaths in this stratum is selected for the survey.

Stratum 2
Cause of death implies that an ELD is possible
Included causes of death: endocrine, nutritional and metabolic diseases; mental and behavioural disorders; diseases of the nervous system; diseases of the respiratory system; diseases of the digestive system; diseases of the genitourinary system (ICD-10 codes: E, F, G, J, K, N).
One out of every four deaths in this stratum is selected for the survey.

Stratum 3
Cause of death implies that an ELD is improbable
All remaining causes of death are included in this stratum (ICD-10 codes: A, D50–D99, H, I, L, M, Q, R, S, T, U, V, Y).
One out of every eight deaths in this stratum is selected for the survey.

* Causes of death were grouped into strata based on the probability of an ELD as observed in the Flemish part of the EURELD six nations study (2001) [14].

** Although there is no box to specify euthanasia in the death certificate, it is occasionally written down by the certifying physician in the section 'immediate cause of death'.

Because end-of-life decision making in minors (1–17 years of age at death) may differ from that in adults, we also integrated a fifth stratum. As there are relatively few deaths of minors annually, all deaths of minors in the period June-November 2007 are sampled to guarantee reliable incidence estimates for deaths in this age category.

### Questionnaire (see additional file 1)

In developing the questionnaire, attention was paid to issues of length, difficulty, clarity, term ambiguity and similarity of content to questionnaires in previous ELD studies. We developed a questionnaire which drew on those of the previous studies in Belgium, the Netherlands and other European countries, the first of which had been developed for the 1990 Dutch survey on ELDs [9]. We used the same set of key questions to ask about the medical decisions that were made at the end of life, thereby making possible incidence estimates comparable to those in earlier studies. Secondary questions regarding the decision-making process preceding an ELD, treatments and care provided, pain and other symptoms present in the last 24 hours before death and the perceived quality of dying were altered or added. The questionnaire was thoroughly analysed and tested by several physicians to correct for any imperfections or ambiguities. Its length was limited to five pages and the difficulty of the questions was kept as low as possible, bearing in mind the complexity of the research subject. The original Flemish version of the questionnaire is provided as additional file 1 to this manuscript.

**Figure 1:**
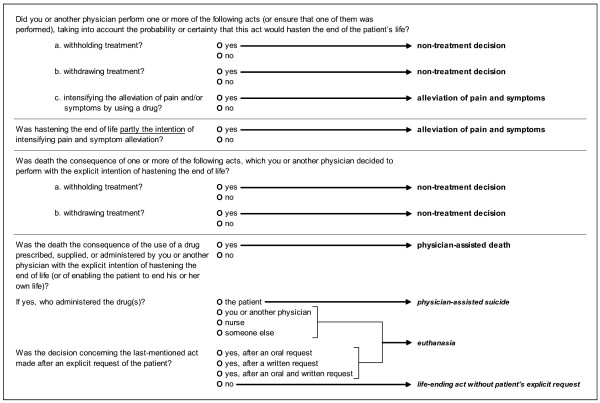
Questions to determine end-of-life decisions.

There are four sections to the questionnaire. In the first, general section the physicians fill in their occupation (general practitioner or specialist), whether they had contact with the patient before his or her death and whether or not the death was sudden and unexpected. The other sections are to be completed only if the treating physician had contact with the patient prior to death and death was not sudden and completely unexpected. The second section asks key questions concerning the medical decisions that were made at the end of the patient's life. Terms such as 'euthanasia' or 'physician-assisted suicide' are not used, as they are emotionally charged and subject to ambiguous and multidimensional definition. Instead, the types of ELDs are more validly determined by establishing (1) what act the physician initiated, (2) to which extent the physician intended life-shortening when initiating the act, and (3) if there had been an explicit request from the patient to initiate the act. Figure 1 shows how a classification of ELDs is derived from the answers to the key questions. If more than one ELD was made, the decision with the most explicit intention of hastening death is given priority in the classification. And if there was more than one act with a similar intention to hasten death, the administering of drugs is chosen over the withholding or withdrawal of treatment. In the third section physicians can note the likely degree to which life was actually shortened and some characteristics of the decision-making process. We included additional questions also posed in the 2005 Dutch ELD study in this section: one about whether or not euthanasia cases were reported, as is required by the Euthanasia Law, and if not why they were not reported and another concerning the term physicians would use to describe their act. The fourth section comprises questions about the characteristics of care and treatment provided at the end of the patient's life, the symptoms observed in the last 24 hours as well as the perceived quality of the patient's death. Finally we integrated a set of questions in this section about palliative or terminal sedation (defined as continuous deep sedation until death). In addition to the types of drugs used for the sedation, the length of the sedation, and the withdrawal of food and fluids, the questionnaire asks about the presence of an explicit request by the patient or the family, possible alternatives to sedation and whether a life-shortening intention was present. Thus, the questionnaire can pursue the paramount question of whether sedation is performed as a treatment decision with no life-shortening intention whatsoever or as an ELD or even as an alternative to euthanasia, as suggested in the literature [7,33-35] and in recent research [20].

### Mailing procedure and anonymity

**Figure 2:**
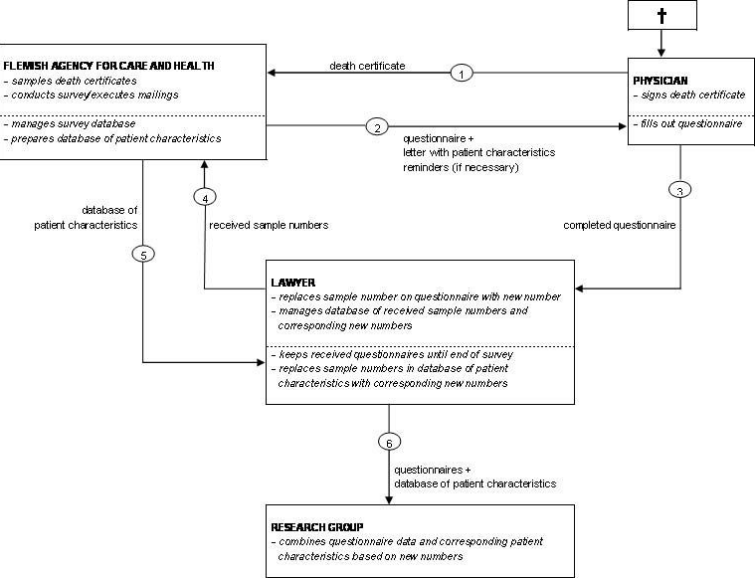
Schematic overview of the mailing and anonymity procedure.

Safeguarding the anonymity of physicians and patients is not only necessary for obvious ethical reasons but also for judicial reasons. Some life-ending acts can be deemed unacceptable by the Belgian criminal law and if anonymity were not guaranteed physicians could risk criminal prosecution for end-of-life decisions reported in this study. Moreover, the response rate to the questionnaire as well as the reliability of the answers will only improve if physicians feel safe enough to answer. Therefore a rigorous procedure was implemented to guarantee that no completed questionnaire could be linked to a particular patient or physician and that both patients and physicians remained anonymous. This procedure has been used in past studies on ELDs, and has proved effective [9,10,13,14,20]. To meet the requirement of anonymity, the different stages of the survey i.e. the sampling and mailing, receiving and processing of the questionnaires are spatially separated. Each stage is performed by different persons. Four parties are involved in the survey, each with specific functions. For a schematic overview of the procedure, see Figure 2.

#### 1. Flemish Agency for Care and Health (of the Flemish Ministry for Welfare, Public Health and Family)

The Flemish Agency for Care and Health, the official department for processing death certificates, is responsible for the sampling of the death certificates, management of the sample database, and the mailing of the questionnaires. Each case is ascribed a unique sample number which is derived from the death certificate number using a fixed algorithm. These sample numbers are used at the end of the study to link the questionnaires to the patients' socio-demographic and morbidity data, derived from the death certificates, in a database provided by the Flemish Agency for Care and Health (cfr. infra). An accompanying letter is included with the questionnaire providing the physician with enough patient characteristics derived from the death certificates to identify the patient (i.e. sex, date of birth, date of death, and municipality of death). The researchers do not have access to the sample database as it contains identifying information of the patients and physicians.

#### 2. Physicians

After identifying the patient by the patient characteristics in the accompanying letter, the physicians can fill out the questionnaires. They are advised to destroy the accompanying letter afterwards. No combination of answers given in the questionnaires can lead to identification of the patient or of the physician.

In some cases, the certifying physician was not the treating physician for the patient in question. In such cases the physician is given a directive to transmit the questionnaire to the treating physician. If the identity of the treating physician is not known the case in question is discarded as being impossible to study. Also, some physicians no longer work at the hospital or practice where the patient died and can therefore not identify the patient or do not have access to the patient file. These cases are also removed from the sample.

#### 3. Lawyer

The completed questionnaires are not returned to the Flemish Agency for Care and Health or to the researchers but instead to a sworn lawyer who is bound to professional confidentiality. The lawyer safeguards the anonymity of the questionnaires received by removing any possible identifying information from them such as notes, stamps and signatures. He also removes the sample numbers and reports them to the Flemish Agency for Care and Health. These cases are subsequently deleted from the sample database so that the certifying physician does not receive further reminders regarding this particular death.

As removing the sample numbers from the questionnaires would make it impossible to link them to the corresponding patient's socio-demographic and morbidity data at the end of the study, the lawyer ascribes a new number to every questionnaire and keeps a database in which the original sample numbers and the corresponding numbers are linked to one another.

The lawyer keeps the received questionnaires until the end of the survey. Afterwards, the Flemish Agency for Care and Health transmits the database of the patients' socio-demographic and morbidity characteristics to the lawyer. The lawyer links the cases in this database to their corresponding new numbers via the original sample numbers and then deletes the original sample numbers. When these sample numbers (derived from the death certificates) are deleted, the information in the database of patient characteristics and the information in the questionnaires can no longer be traced back to the corresponding death certificates.

#### 4. Research group

After this complex procedure, the information in the database and questionnaires is strictly anonymous: the replacement of sample numbers by new numbers cuts the link between questionnaires and death certificates and neither the combination of patient characteristics nor the information provided in the questionnaires can lead to the identification of patients or physicians. The lawyer can thus transmit the questionnaires and the database with patient characteristics to the researchers, who combine the data from both (using the new numbers) into one database for analysis.

### Total Design Method (TDM)

All efforts to attain a representative sample of deaths are ineffective if the response rate does not reach a minimal level, therefore some measures are taken to achieve this. We followed some prescriptions from Don A. Dillman's Total Design Method (TDM) for mail surveys [36]. Firstly, we established an intensive follow-up mailing. After the questionnaire is sent out, the physician receives a maximum of three reminders at an interval of 14 days until the questionnaire is returned. In the second reminder a new copy of the questionnaire is included, thus anticipating the possibility of the physician having lost the original.

The TDM is based on the costs-benefits analysis of social action; in a social context a person only acts if there are advantages in that action. Given this principle, the probability of participating in a study will be greater if the study succeeds in keeping the costs (i.e. disadvantages, efforts, time) of participation as low as possible while at the same time maximising the gains for the respondent [36]. To minimise the costs of participation, the questionnaire was kept as short as possible, and the difficulty-level of the questions and answer options as low as possible considering the complexity of the study subject. A maximum of five death cases per physician was decided on to limit responder fatigue. A stamped return envelope was included with every questionnaire sent out. We feel that guaranteeing the anonymity of physicians and patients is also an important measure in minimising the potential costs of participation. To maximise the gains for the physicians, we stress the importance of the study for the medical field, as the results can contribute to better and more effective policies on end-of-life decisions in Belgium. Participation can thus ultimately result in better conditions for physicians to work in, as well as for patients at the end of their lives. We also deem it important to communicate the results of the study to the participants. All participating physicians are assured of an invitation to a seminar on the study after the data collection. As an extra incentive, a valuable artwork will be awarded to a randomly chosen participating physician. Due to the large number of participating physicians, the funds are not sufficient to reward every individual physician financially.

Additionally to the involvement of the Vrije Universiteit Brussel and Ghent University in conducting the study, representatives of two other Flemish universities, the Katholieke Universiteit Leuven and Universiteit Antwerpen, and the Scientific Institute of Public Health support the study to increase its visibility. Also, the positive recommendation of the Belgian National Disciplinary Board of Physicians (cfr. infra) is mentioned in the accompanying letter.

### Non-response survey

After the data collection a one-page questionnaire is sent to all non-responding physicians, asking about their reasons for non-response. Besides providing interesting information on non-response in general, these reasons can warrant the removal of some cases from the sample because of the physician's inability to fill out the questionnaire (e.g. the patient cannot be identified with the information provided, the physician no longer has access to the medical file, the certifying physician is not the attending physician and can not identify him or her, or the physician never received the questionnaire).

### Data analysis

The researchers will prepare an SPSS 16.0 (SPSS Inc.) database with coding scheme for a certified data management company which will enter the data. Range and skip checks will prevent key-punching errors, and the data quality will further be improved partly via double data-entry and partly through extensive random sample checks. The researchers will perform data cleaning via SPSS syntax operations.

The data will be weighted to correct for the disproportionate stratification of underlying causes of death and the deaths of minors. The influence of non-response on the representativity of the data will subsequently be checked and weighted through a comparison of proportionality of underlying causes of death and other patient characteristics (i.e. sex, age, educational level, marital status, living situation, province of residence, month of death and place of death) between deaths where responses have been received and deaths within the general population in 2007.

Data will be analysed with descriptive statistics (valid percentages and 95% confidence intervals), as well as bi- and multivariate association statistics using SPSS version 16.0.

### Recommendations

Positive recommendations for the anonymity procedure and study protocol were obtained from the Ethical Review Board of the University Hospital of the Vrije Universiteit Brussel, the Ethics Committee of the University Hospital of Ghent University, the Belgian National Disciplinary Board of Physicians and the Belgian Federal Privacy Commission.

## Discussion

The 2007 Flemish ELD study aims to produce representative incidence estimates of end-of-life decisions in Flanders and to describe their characteristics as well as the circumstances under which they occur. A summary of the key characteristics of the study design is shown in Table 2.

**Table 2:** Summary of the study design

DEATH CERTIFICATE SURVEY
✓ large sample of deaths
✓ nationwide (over care settings and causes of death)
✓ stratified disproportionately based on cause of death
QUESTIONNAIRE
✓ short and validated questionnaire
✓ key questions of ELDs identical to those in earlier studies
✓ emotionally charged terms absent in key questions

MAILING PROCEDURE
✓ guarantee of anonymity for physicians and patients
✓ response-increasing measures
✓ intensive follow-up mailing

The four methodological challenges formulated at the outset of this article were addressed: (1) we opted for a retrospective study design based on death certificates, as this design provides the best chances of obtaining reliable incidence estimates of ELDs and their characteristics from a large and representative sample of deaths. Moreover, the disproportionate stratification based on the likelihood of an ELD preceding death further increases the statistical power of the results (2) comparability of the data to earlier studies is ensured by using the same set of key questions in the questionnaire, and by keeping the main characteristics of the study design constant (3) a rigorous procedure involving a lawyer as intermediary between physicians and researchers is employed to guarantee the anonymity of physicians and patients and (4) we use several measures from the Total Design Method to obtain a satisfactory response rate.

The study has some strengths as well as weaknesses related to the use of death certificates and the study design in general.

### Strengths

Most studies in end-of-life care research are limited with regard to sample size, care settings or illness types. This impedes the chances of obtaining representative population data in end-of-life care research. For example, one study examined end-of-life practices in a sample of dying patients but was set only in intensive care units [16]. Using death certificates on the other hand facilitates the obtaining of robust data for the entire population, as a large sample of deaths can be drawn across care settings including all causes of death [28,37]. And, because of its nationwide scope, this study design is most suitable for international comparative research, as the EURELD six nations study has shown [14]. Also, the retrospective nature of the study design does not encounter the problems of patient burden, attrition or non-response of the sickest patients found in prospective study designs [28,29], and it does not run the risk of influencing end-of-life practices, which is a realistic possibility in prospective studies.

Because all deaths must be reported to the proper government authorities, death certificates also allow the use of the death as the unit of measurement, providing a clear denominator for reliable estimation of the incidence of ELDs [28]. The reliability of these estimates is not guaranteed in studies based on the last deceased patient treated by a representative sample of physicians [11,12,18], as the unit of measurement in these studies is the physician and the number of deaths preceded by an ELD is estimated on the basis of physician characteristics. Moreover, in contrast to the death certificate design, physicians in these studies are not guaranteed to have attended a death.

Using death certificates also facilitates the anonymous linking of patient characteristics to the information provided in the questionnaires, allowing the study of associations between socio-demographic and morbidity characteristics of the patient on the one hand, and end-of-life decision making and provided care at the end of life on the other hand [38].

Another strength of the present study is that, whereas in other end-of-life research physicians can be inadvertently selected on the basis of their interest in or attitudes towards end-of-life practices, the use of death certificates excludes the possibility of a biased selection of physicians.

### Weaknesses

The physician signing the death certificate is occasionally not the patient's treating physician, and therefore is not in a position to fill out the questionnaire. Despite the directive to transmit the questionnaire to the treating physician, some cases are impossible to study as the treating physician cannot be identified. In some instances not even the identity of the patient can be retrieved because the treating physician no longer has access to the patient file.

Because death certificates have to be processed by the proper authorities before they can be made available for research, there can be a considerable delay between the patient's death and the study of that death [37]. The delay in our study has reached as much as four months (there is variation across countries, ranging from two to six months). We can therefore not exclude some influence of recall bias. To address this issue, we encourage physicians to fill in their questionnaires using the patient files, which are mostly readily at their disposal.

Given the death as the unit of measurement and the large number of deaths studied, one physician can receive several questionnaires. Despite a maximum of five cases for each physician, responder fatigue and diminishing response rates can result.

A structured and semi-closed questionnaire can often overlook the intricacies of certain end-of-life decisions. Moreover, the questionnaire used in this study has, for reasons of response, been limited in length and time-consuming questions have been left out. There is a risk that in some cases vital information can be missed. To counter this problem, a section is provided at the end of the questionnaire in which the physician can comment or elaborate on the answers given.

### Opportunities for future research

The present study is the third in a series of death certificate studies in Belgium. Keeping the study design and the questionnaire constant creates the opportunity for future studies to build on the comparable data obtained in the past and to identify accurately developments in the field of ELDs. The study design can be applied to research in other countries, so that data can be produced for international comparative research. Comparable data are already available in Belgium, the Netherlands, Italy, Switzerland, Denmark and Sweden [8-10,13-15,19,20]. Furthermore, the use of death certificates in end-of-life care research need not be limited to ELDs; they can also be applied in retrospective research on other issues in this field [37].

## Abbreviations

(Medical) end-of-life decisions: ELDs; Total Design Method: TDM; Monitoring quality of End-of-Life Care: MELC study.

## Competing interests

The authors declare that they have no competing interests.

## Authors' contributions

All authors contributed to the design and conceptual framework of the protocol. The manuscript was drafted by KC, with further input from all other authors. LD and FM are the project supervisors. All authors read, revised and approved the final manuscript.

## Pre-publication history

The pre-publication history for this paper can be accessed here:



## Supplementary Material

Additional file 1 Questionnaire of the 2007 Flemish ELD study. Click here for file
