# Supplementary material for: A post-mortem survey on end-of-life decisions using a representative sample of death certificates in Flanders, Belgium: research protocol
Source: BMC Public Health. 2008 Aug 27;8:299. doi: 10.1186/1471-2458-8-299 (PMC2533325; doi:10.1186/1471-2458-8-299)
Supplement: Additional file 1 — Questionnaire of the 2007 Flemish ELD study. [file 1471-2458-8-299-S1.doc]

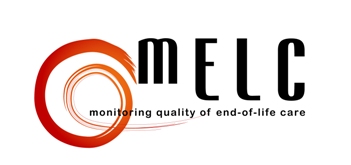


| ***Medische beslissingen***  ***rond het levenseinde*** | ***2007*** |
| --- | --- |

| ***Algemeen*** | | | | | | | |
| --- | --- | --- | --- | --- | --- | --- | --- |
|  |  | | | | | | |
| 1. U was met betrekking tot dit sterfgeval werkzaam als ... |  | | specialist (of specialist in opleiding) | | | | |
|  | welk specialisme? | | | | |  |  |
|  |  | | huisarts (of huisarts in opleiding) | | | | |
|  |  | | andere |  | | |  |
|  |  | | | | | | |
| 1. Van wanneer dateerde uw eerste contact met de patiënt? |  | | vóór of tijdens het overlijden | | | | |
|  |  | | na het overlijden 🡺 **door naar vraag 29** | | | | |
|  |  | | | | | | |
| 1. Ging het om een plotseling en geheel onverwacht overlijden? |  | | ja 🡺 **door naar vraag 27** | | | | |
|  |  | | neen | | | | |
|  |  | | | | | | |
| ***Medische handelwijzen*** | | | | | | | |
|  | | | | | | | |
| 1. Heeft u of een andere arts één of meer van de volgende handelwijzen uitgevoerd of doen uitvoeren, rekening houdend met de mogelijkheid dat deze handelwijze het levenseinde van de patiënt zou bespoedigen? *– zowel* ***a****,* ***b*** *als* ***c*** *beantwoorden –* | | | | | | | |
|  | | | | | | | |
|  |  | | | | | | |
| 1. Het niet instellen van een behandeling*? |  | | ja | | | | |
|  |  | | neen | | | | |
|  |  | | | | | | |
| Zo ja, welke behandeling(en) betrof dit? |  |  | | | | |  |
|  |  | | | | | | |
|  |  |  | | | | |  |
|  |  | | | | | | |
| 1. Het staken van een behandeling*? |  | | ja | | | | |
|  |  | | neen | | | | |
|  |  | | | | | | |
| Zo ja, welke behandeling(en) betrof dit? |  |  | | | | |  |
|  |  | | | | | | |
|  |  |  | | | | |  |
|  |  | | | | | | |
| 1. Het intensiveren van pijn- en/of symptoombestrijding |  | | ja | | | | |
| d.m.v. één of meer middelen? |  | | neen 🡺 **door naar vraag 6** | | | | |
|  |  | | | | | | |
| Zo ja, welk(e) middel(en) werd(en) gebruikt? |  | | morfine of ander opiaat | | | | |
| *– meerdere antwoorden mogelijk –* |  | | benzodiazepine | | | | |
|  |  | | ander middel | |  | |  |
| ** onder ‘behandeling’ wordt ook de kunstmatige toediening van*  *vocht en/of voeding verstaan.* |  | | | | | | |
|  |  | | | | | | |
| 1. Was het bespoedigen van het levenseinde mede het doel |  | | ja | | | | |
| van het intensiveren van pijn- en/of symptoombestrijding? |  | | neen | | | | |
|  |  | | | | | | |
|  | | | | | | | |
| 1. Was het overlijden het gevolg van één of meer van de volgende handelwijzen, waartoe door u of een andere arts is besloten met het uitdrukkelijke doel het levenseinde van de patiënt te bespoedigen? *– zowel* ***a*** *als* ***b*** *beantwoorden –* | | | | | | | |
|  | | | | | | | |
|  |  | | | | | | |
| - - 1. Het niet instellen van een behandeling*? |  | | ja | | | | |
|  |  | | neen | | | | |
|  |  | | | | | | |
| Zo ja, welke behandeling(en) betrof dit? |  |  | | | | |  |
|  |  | | | | | | |
|  |  |  | | | | |  |
|  |  | | | | | | |
| 1. Het staken van een behandeling*? |  | | ja | | | | |
|  |  | | neen | | | | |
|  |  | | | | | | |
| Zo ja, welke behandeling(en) betrof dit? |  |  | | | | |  |
|  |  | | | | | | |
|  |  |  | | | | |  |
| ** onder ‘behandeling’ wordt ook de kunstmatige toediening van*  *vocht en/of voeding verstaan.* |  | | | | | | |
|  |  | | | | | | |

|  |  | | | | | | | |
| --- | --- | --- | --- | --- | --- | --- | --- | --- |
| 1. Was het overlijden het gevolg van het gebruik van een |  | | ja | | | | | |
| middel dat door u of een andere arts werd voor-geschreven, verstrekt of toegediend met het |  | | neen | | | | | |
| uitdrukkelijke doel het levenseinde van de patiënt te |  | | | | | | | |
| bespoedigen (of de patiënt in staat te stellen zelf het |  | | | | | | | |
| leven te beëindigen)? |  | | | | | | | |
|  |  | | | | *Naam* | *Toedieningswijze* | *Dosis* |  |
| Zo ja, welk(e) middel(en) betrof dit?  *– meerdere antwoorden mogelijk –* |  | spierverslapper (curare  of gelijkaardig middel) | | |  |  |  |  |
|  |  | barbituraat | | |  |  |  |  |
|  |  | benzodiazepine | | |  |  |  |  |
|  |  | morfine of ander opiaat | | |  |  |  |  |
|  |  | ander middel | | |  |  |  |  |
|  |  | | | | | | | |
| Zo ja, door wie is (zijn) dit (deze) middel(en) toegediend? |  | | de patiënt zelf | | | | | |
| *– meerdere antwoorden mogelijk –* |  | | u of een andere arts | | | | | |
|  |  | | verpleegkundige | | | | | |
|  |  | | iemand anders |  | | | |  |
|  |  | | | | | | | |
| Indien het (de) middel(en) niet door een arts werd(en) |  | | ja | | | | | |
| toegediend, was u of een andere arts aanwezig bij de |  | | neen | | | | | |
| toediening? |  | | | | | | | |
|  |  | | | | | | | |

| **Indien ‘ja’ is geantwoord op één van de onderdelen van de vragen 4 tot en met 7 🡺 door naar vraag 8**  **Indien op geen enkel onderdeel van de vragen 4 tot en met 7 ‘ja’ is geantwoord 🡺 door naar vraag 21** |
| --- |

| ***De laatstgenoemde handelwijze*** | | | | |
| --- | --- | --- | --- | --- |
|  | | | | |
| **Let op: de vragen 8 tot en met 20 hebben betrekking op de *laatstgenoemde handelwijze*, dit wil zeggen op het laatst gegeven ‘ja’-antwoord bij de vragen 4 tot en met 7** | | | | |
|  |  | | | |
| 1. Met hoeveel tijd is het leven van de patiënt naar uw schatting |  | meer dan een half jaar | | |
| verkort door de laatstgenoemde handelwijze? |  | één tot zes maanden | | |
|  |  | één tot vier weken | | |
|  |  | één tot zeven dagen | | |
|  |  | minder dan 24 uur | | |
|  |  | heeft waarschijnlijk geen verkorting van de levensduur gegeven | | |
|  |  | | | |
| 1. Heeft u of een andere arts de (mogelijke) bespoediging van |  | ja | | |
| het levenseinde door die laatstgenoemde handelwijze |  | neen 🡺 **door naar vraag 13** | | |
| besproken met de patiënt? |  | | | |
|  |  | | | |
| 1. Achtte u de patiënt tijdens deze bespreking in staat zijn of |  | ja | | |
| haar situatie te overzien en daarover op adequate wijze een |  | neen | | |
| besluit te nemen? |  | | | |
|  |  | | | |
| 1. Is de beslissing over de laatstgenoemde handelwijze |  | ja, na een mondeling verzoek | | |
| genomen na een uitdrukkelijk verzoek van de patiënt? |  | ja, na een schriftelijk verzoek | | |
|  |  | ja, na een mondeling én een schriftelijk verzoek | | |
|  |  | neen 🡺 **door naar vraag 16** | | |
|  |  | | | |
| 1. Achtte u de patiënt tijdens dit verzoek in staat zijn of haar |  | ja 🡺 **door naar vraag 16** | | |
| situatie te overzien en daarover op adequate wijze een |  | neen 🡺 **door naar vraag 16** | | |
| besluit te nemen? |  | | | |
|  |  | | | |
| 1. Achtte u de patiënt in staat zijn of haar situatie te overzien en |  | ja | | |
| daarover op adequate wijze een besluit te nemen? |  | neen | | |
|  |  | | | |
| 1. Om welke reden is de (mogelijke) bespoediging van het |  | de patiënt was te jong | | |
| levenseinde door de laatstgenoemde handelwijze niet met de |  | de patiënt was subcomateus of buiten bewustzijn | | |
| patiënt besproken? |  | de patiënt was dement | | |
| *– meerdere antwoorden mogelijk –* |  | de patiënt was verstandelijk gehandicapt | | |
|  |  | de patiënt had een psychiatrische stoornis | | |
|  |  | de laatstgenoemde handelwijze was duidelijk het beste voor de patiënt | | |
|  |  | de bespreking zou de patiënt meer schaden dan goed doen | | |
|  |  | andere reden |  |  |
|  |  | | | |
| 1. Had de patiënt, voor zover u bekend, ooit een wens tot |  | ja, uitdrukkelijk | | |
| bespoediging van het levenseinde kenbaar gemaakt? |  | ja, maar niet uitdrukkelijk | | |
|  |  | neen | | |
|  |  | | | |

|  | | | | | | |  | | | | | | | | | | | | | | | | | | | | | | | | |
| --- | --- | --- | --- | --- | --- | --- | --- | --- | --- | --- | --- | --- | --- | --- | --- | --- | --- | --- | --- | --- | --- | --- | --- | --- | --- | --- | --- | --- | --- | --- | --- |
| 1. Was er een voorafgaandelijke schriftelijke wilsverklaring van | | | | | | |  | | | ja, een wilsverklaring voor euthanasie | | | | | | | | | | | | | | | | | | | | | |
| de patiënt? | | | | | | |  | | | ja, een andere wilsverklaring | | | | | | | | | | | | | | | | | | | | | |
|  | | | | | | |  | | | neen | | | | | | | | | | | | | | | | | | | | | |
|  | | | | | | |  | | | | | | | | | | | | | | | | | | | | | | | | |
| 1. Heeft u of een andere arts de (mogelijke) bespoediging van | | | | | | |  | | | ja, met collega-arts(en) | | | | | | | | | | | | | | | | | | | | | |
| het levenseinde met anderen besproken voordat werd | | | | | | |  | | | ja, met zorgverlener(s) gespecialiseerd in palliatieve zorg | | | | | | | | | | | | | | | | | | | | | |
| besloten tot de laatstgenoemde handelwijze? | | | | | | |  | | | ja, met verpleegkundige(n) | | | | | | | | | | | | | | | | | | | | | |
| *– meerdere antwoorden mogelijk –* | | | | | | |  | | | ja, met de partner en/of familie van de patiënt | | | | | | | | | | | | | | | | | | | | | |
|  | | | | | | |  | | | ja, met anderen | | | | | | | | | | | | |  | | | | | | |  | |
|  | | | | | | |  | | | neen | | | | | | | | | | | | | | | | | | | | | |
|  | | | | | | |  | | | | | | | | | | | | | | | | | | | | | | | | |
| Indien het werd besproken met collega-artsen: betrof dit ook | | | | | | |  | | | ja, consultatie van een LEIF-arts | | | | | | | | | | | | | | | | | | | | | |
| een consultatie in het kader van de wettelijke voorschriften | | | | | | |  | | | ja, consultatie van een andere arts | | | | | | | | | | | | | | | | | | | | | |
| voor euthanasie? | | | | | | |  | | | neen | | | | | | | | | | | | | | | | | | | | | |
| *– meerdere antwoorden mogelijk –* | | | | | | |  | | | | | | | | | | | | | | | | | | | | | | | | |
|  | | | | | | |  | | | | | | | | | | | | | | | | | | | | | | | | |
| 1. Wat was (waren) de belangrijkste reden(en) om te besluiten | | | | | | |  | | | de patiënt had (ernstige) pijn | | | | | | | | | | | | | | | | | | | | | |
| tot de laatstgenoemde handelwijze? | | | | | | |  | | | de patiënt had andere (ernstige) symptomen | | | | | | | | | | | | | | | | | | | | | |
| *– meerdere antwoorden mogelijk –* | | | | | | |  | | | verzoek of wens van de patiënt | | | | | | | | | | | | | | | | | | | | | |
|  | | | | | | |  | | | verzoek of wens van de partner en/of familie | | | | | | | | | | | | | | | | | | | | | |
|  | | | | | | |  | | | verwacht (verder) lijden van de patiënt | | | | | | | | | | | | | | | | | | | | | |
|  | | | | | | |  | | | er was geen uitzicht op verbetering | | | | | | | | | | | | | | | | | | | | | |
|  | | | | | | |  | | | het leven niet onnodig verlengen | | | | | | | | | | | | | | | | | | | | | |
|  | | | | | | |  | | | geringe verwachte levenskwaliteit | | | | | | | | | | | | | | | | | | | | | |
|  | | | | | | |  | | | situatie werd ondraaglijk voor de naasten | | | | | | | | | | | | | | | | | | | | | |
|  | | | | | | |  | | | verlies van waardigheid | | | | | | | | | | | | | | | | | | | | | |
|  | | | | | | |  | | | andere reden, desgewenst toelichten bij vraag 29 | | | | | | | | | | | | | | | | | | | | | |
|  | | | | | | |  | | | | | | | | | | | | | | | | | | | | | | | | |
| 1. Welke term past volgens u het best bij de laatstgenoemde | | | | | | |  | | | niet-behandelbeslissing | | | | | | | | | | | | | | | | | | | | | |
| handelwijze? | | | | | | |  | | | symptoombestrijding | | | | | | | | | | | | | | | | | | | | | |
| *– slechts één antwoord mogelijk –* | | | | | | |  | | | palliatieve of terminale sedatie | | | | | | | | | | | | | | | | | | | | | |
|  | | | | | | |  | | | levensbeëindiging uit compassie | | | | | | | | | | | | | | | | | | | | | |
|  | | | | | | |  | | | euthanasie | | | | | | | | | | | | | | | | | | | | | |
|  | | | | | | |  | | | hulp bij zelfdoding | | | | | | | | | | | | | | | | | | | | | |
|  | | | | | | |  | | | andere | | | | | |  | | | | | | | | | | | | | |  | |
|  | | | | | | |  | | | | | | | | | | | | | | | | | | | | | | | | |
| 1. Heeft u of een andere arts de laatstgenoemde handelwijze | | | | | | |  | | | ja 🡺 **door naar vraag 21** | | | | | | | | | | | | | | | | | | | | | |
| gemeld aan de controle- en evaluatiecommissie voor | | | | | | |  | | | neen | | | | | | | | | | | | | | | | | | | | | |
| euthanasie? | | | | | | |  | | | | | | | | | | | | | | | | | | | | | | | | |
|  | | | | | | |  | | | | | | | | | | | | | | | | | | | | | | | | |
| Om welke reden(en) niet? | | | | | | |  | | | het betrof geen euthanasie | | | | | | | | | | | | | | | | | | | | | |
| *– meerdere antwoorden mogelijk –* | | | | | | |  | | | melden geeft te veel rompslomp | | | | | | | | | | | | | | | | | | | | | |
|  | | | | | | |  | | | euthanasie is een zaak tussen arts en patiënt | | | | | | | | | | | | | | | | | | | | | |
|  | | | | | | |  | | | er was mogelijk niet aan alle zorgvuldigheidseisen voldaan | | | | | | | | | | | | | | | | | | | | | |
|  | | | | | | |  | | | vanwege mogelijke juridische consequenties | | | | | | | | | | | | | | | | | | | | | |
|  | | | | | | |  | | | andere reden | | | | | | | | |  | | | | | | | | | |  | | |
|  | | | | | | |  | | | | | | | | | | | | | | | | | | | | | | | | |
| ***Zorg en behandeling*** | | | | | | | | | | | | | | | | | | | | | | | | | | | | | | | |
|  | | | | | | |  | | | | | | | | | | | | | | | | | | | | | | | | |
| 1. Hoe lang is de patiënt in behandeling geweest voor de | | | | | | |  | | | één tot zeven dagen | | | | | | | | | | | | | | | | | | | | | |
| aandoening die tot zijn of haar overlijden heeft geleid? | | | | | | |  | | | één tot vier weken | | | | | | | | | | | | | | | | | | | | | |
|  | | | | | | |  | | | één tot drie maanden | | | | | | | | | | | | | | | | | | | | | |
|  | | | | | | |  | | | drie tot zes maanden | | | | | | | | | | | | | | | | | | | | | |
|  | | | | | | |  | | | zes maanden tot een jaar | | | | | | | | | | | | | | | | | | | | | |
|  | | | | | | |  | | | meer dan een jaar | | | | | | | | | | | | | | | | | | | | | |
|  | | | | | | |  | | | | | | | | | | | | | | | | | | | | | | | | |
| 1. Waar was de behandeling tijdens de laatste week vóór het | | | | | | |  | | | genezing | | | | | | | | | | | | | | | | | | | | | |
| overlijden in hoofdzaak op gericht? | | | | | | |  | | | levensverlenging | | | | | | | | | | | | | | | | | | | | | |
|  | | | | | | |  | | | comfort | | | | | | | | | | | | | | | | | | | | | |
|  | | | | | | |  | | | | | | | | | | | | | | | | | | | | | | | | |
|  | | | | | | | | | | | | | | | | | | | | | | | | | | | | | | | |
| 1. In welke mate waren naar uw schatting de volgende symptomen of verschijnselen bij de patiënt aanwezig   tijdens de laatste 24 uur vóór het overlijden – ondanks eventuele behandeling? | | | | | | | | | | | | | | | | | | | | | | | | | | | | | | | |
|  | | | | | | | | | | | | | | | | | | | | | | | | | | | | | | | |
|  | | | | | | | | | | | | | | | | | | | | | | | | | | | | | | | |
|  | 0 | 1 | 2 | 3 | 4 | 5 | 6 | | | | 7 | 8 | | | 9 | | | 10 | | | |  | | | | | | | | | |
| geen pijn |  |  |  |  |  |  |  | | | |  |  | | |  | | |  | | | | ergst mogelijke pijn | | | | | | | | | |
| niet vermoeid |  |  |  |  |  |  |  | | | |  |  | | |  | | |  | | | | ergst mogelijke vermoeidheid | | | | | | | | | |
| niet misselijk |  |  |  |  |  |  |  | | | |  |  | | |  | | |  | | | | ergst mogelijke misselijkheid | | | | | | | | | |
| niet depressief |  |  |  |  |  |  |  | | | |  |  | | |  | | |  | | | | ergst mogelijke depressie | | | | | | | | | |
| niet angstig |  |  |  |  |  |  |  | | | |  |  | | |  | | |  | | | | ergst mogelijke angst | | | | | | | | | |
| niet suf |  |  |  |  |  |  |  | | | |  |  | | |  | | |  | | | | ergst mogelijke sufheid | | | | | | | | | |
| best mogelijke eetlust |  |  |  |  |  |  |  | | | |  |  | | |  | | |  | | | | slechtst mogelijke eetlust | | | | | | | | | |
| best mogelijk gevoel van welbevinden |  |  |  |  |  |  |  | | | |  |  | | |  | | |  | | | | slechtst mogelijk gevoel van welbevinden | | | | | | | | | |
| niet kortademig |  |  |  |  |  |  |  | | | |  |  | | |  | | |  | | | | ergst mogelijke kortademigheid | | | | | | | | | |
| bij bewustzijn |  |  |  |  |  |  |  | | | |  |  | | |  | | |  | | | | comateus | | | | | | | | | |
| niet verward |  |  |  |  |  |  |  | | | |  |  | | |  | | |  | | | | ergst mogelijke verwardheid | | | | | | | | | |
|  |  | | | | | | | | | | | | | | | | | | | | |  | | | | | | | | | |
|  | | | | | | | | | | | | | | | | | | | | | | | | | | | | | | | |
|  | | | | | | |  | | | | | | | | | | | | | | | | | | | | | | | | |
| 1. Werd de patiënt tot aan het overlijden continu in diepe | | | | | | |  | | | ja | | | | | | | | | | | | | | | | | | | | | |
| sedatie of coma gehouden d.m.v. één of meer middelen? | | | | | | |  | | | neen 🡺 **door naar vraag 25** | | | | | | | | | | | | | | | | | | | | | |
|  | | | | | | |  | | | | | | | | | | | | | | | | | | | | | | | | |
| Welk(e) middel(en) werd(en) daartoe gebruikt? | | | | | | |  | | | midazolam | | | | | | | | | | | | | | | | | | | | | |
| *– meerdere antwoorden mogelijk –* | | | | | | |  | | | ander benzodiazepine | | | | | | | | | | | | | | | | | | | | | |
|  | | | | | | |  | | | morfine of ander opiaat | | | | | | | | | | | | | | | | | | | | | |
|  | | | | | | |  | | | ander middel | | | | | | | | | |  | | | | | | | | | |  | |
|  | | | | | | |  | | | | | | | | | | | | | | | | | | | | | | | | |
| Hoe lang vóór het overlijden werd gestart met het continu | | | | | | |  |  | | | | | uren | | | | | | | | | | | | | | | | | | |
| diep sederen van de patiënt? | | | | | | |  |  | | | | | dagen | | | | | | | | | | | | | | | | | | |
|  | | | | | | |  |  | | | | | weken | | | | | | | | | | | | | | | | | | |
|  | | | | | | |  | | | | | | | | | | | | | | | | | | | | | | | | |
| Kreeg de patiënt daarbij kunstmatig vocht en/of voeding | | | | | | |  | | | ja, continu tot aan het overlijden | | | | | | | | | | | | | | | | | | | | | |
| toegediend? | | | | | | |  | | | ja, maar niet tot aan het overlijden | | | | | | | | | | | | | | | | | | | | | |
|  | | | | | | |  | | | neen | | | | | | | | | | | | | | | | | | | | | |
|  | | | | | | |  | | | | | | | | | | | | | | | | | | | | | | | | |
| Is de beslissing over het continu diep sederen genomen | | | | | | |  | | | ja, met instemming van de patiënt | | | | | | | | | | | | | | | | | | | | | |
| met instemming en/of op verzoek van de patiënt? | | | | | | |  | | | ja, op verzoek van de patiënt | | | | | | | | | | | | | | | | | | | | | |
| *– meerdere antwoorden mogelijk –* | | | | | | |  | | | neen | | | | | | | | | | | | | | | | | | | | | |
|  | | | | | | |  | | | | | | | | | | | | | | | | | | | | | | | | |
| Is de beslissing over het continu diep sederen genomen | | | | | | |  | | | ja, met instemming van de naasten | | | | | | | | | | | | | | | | | | | | | |
| met instemming en/of op verzoek van de naasten? | | | | | | |  | | | ja, op verzoek van de naasten | | | | | | | | | | | | | | | | | | | | | |
| *– meerdere antwoorden mogelijk –* | | | | | | |  | | neen | | | | | | | | | | | | | | | | | | | | | | |
|  | | | | | | |  | | | | | | | | | | | | | | | | | | | | | | | | |
| Waren er naast continue diepe sedatie alternatieven om | | | | | | |  | | | neen | | | | | | | | | | | | | | | | | | | | | |
| de symptomen te behandelen? | | | | | | |  | | | ja, symptoombestrijding zonder continue diepe sedatie | | | | | | | | | | | | | | | | | | | | | |
| *– meerdere antwoorden mogelijk –* | | | | | | |  | | | ja, maar alleen levensbeëindiging | | | | | | | | | | | | | | | | | | | | | |
|  | | | | | | |  | | | ja, andere | | | | | | |  | | | | | | | | | | | | | |  |
|  | | | | | | |  | | | | | | | | | | | | | | | | | | | | | | | | |
| Deze wijze van diep sederen, al dan niet in combinatie met | | | | | | |  | | | wetende dat dit het levenseinde niet zou bespoedigen | | | | | | | | | | | | | | | | | | | | | |
| het kunstmatig toedienen van vocht en/of voeding, werd uitgevoerd ... | | | | | | |  | | | rekening houdend met de mogelijke bespoediging van het levenseinde | | | | | | | | | | | | | | | | | | | | | |
|  | | | | | | |  | | | mede met het doel het levenseinde te bespoedigen | | | | | | | | | | | | | | | | | | | | | |
|  | | | | | | |  | | | met het uitdrukkelijke doel het levenseinde te bespoedigen | | | | | | | | | | | | | | | | | | | | | |
|  | | | | | | |  | | | | | | | | | | | | | | | | | | | | | | | | |
| 1. Heeft de patiënt morfine en/of een ander opiaat toegediend | | | | | | |  | | | ja | | | | | | | | | | | | | | | | | | | | | |
| gekregen tijdens de laatste 24 uur vóór het overlijden? | | | | | | |  | | | neen 🡺 **door naar vraag 26** | | | | | | | | | | | | | | | | | | | | | |
|  | | | | | | |  | | | | | | | | | | | | | | | | | | | | | | | | |
|  | | | | | | |  | | | | | | | | | | *Middel* | | | | | | | | | | *Dosering*  *(laatste 24 uur)* | | | | |
| Naam van het (de) middel(en) en dosering in | | | | | | | Pleisters | | | | | | |  | | | fentanyl (o.a. Durogesic®) | | | | | | | | | |  | µg/uur | | | |
| de laatste 24 uur vóór het overlijden? | | | | | | | Pomp | | | | | | |  | | | morfine | | | | | | | | | |  | mg | | | |
| *– meerdere antwoorden mogelijk –* | | | | | | | Injecties | | | | | | |  | | | morfine | | | | | | | | | |  | mg | | | |
|  | | | | | | |  | | | | | | |  | | | piritramide (o.a. Dipidolor®) | | | | | | | | | |  | mg | | | |
|  | | | | | | | Zetpillen | | | | | | |  | | | morfine | | | | | | | | | |  | mg | | | |
|  | | | | | | | Drank | | | | | | |  | | | morfine | | | | | | | | | |  | mg | | | |
|  | | | | | | |  | | | | | | |  | | | methadon | | | | | | | | | |  | mg | | | |
|  | | | | | | | Tabletten | | | | | | |  | | | morfine retard (o.a. MS Contin®) | | | | | | | | | |  | mg | | | |
|  | | | | | | |  | | | | | | |  | | | morfine | | | | | | | | | |  | mg | | | |
|  | | | | | | |  | | | | | | |  | | | tramadol (o.a. Tramal®) | | | | | | | | | |  | mg | | | |
|  | | | | | | |  | | | | | | |  | | | oxycodon (o.a. Oxycontin®) | | | | | | | | | |  | mg | | | |
|  | | | | | | | Druppels | | | | | | |  | | | tramadol (o.a. Tramal®) | | | | | | | | | |  | mg | | | |
|  | | | | | | | Anders | | | | | | |  | | | middel | | | | | | |  | | |  | mg | | | |
|  | | | | | | |  | | | | | | | | | | toedieningswijze | | | | | | | |  | |  | | | | |
|  | | | | | | |  | | | | | | | | | | | | | | | | | | | | | | | | |
| Is een hogere dosis gegeven dan nodig was om pijn en/of | | | | | | |  | | | ja | | | | | | | | | | | | | | | | | | | | | |
| andere symptomen te bestrijden? | | | | | | |  | | | neen | | | | | | | | | | | | | | | | | | | | | |
|  | | | | | | |  | | | | | | | | | | | | | | | | | | | | | | | | |
| Hoe lang vóór het overlijden werd gestart met het toedienen | | | | | | |  |  | | | | | uren | | | | | | | | | | | | | | | | | | |
| van de morfine en/of een ander opiaat? | | | | | | |  |  | | | | | dagen | | | | | | | | | | | | | | | | | | |
|  | | | | | | |  |  | | | | | weken | | | | | | | | | | | | | | | | | | |
|  | | | | | | |  | | | | | | | | | | | | | | | | | | | | | | | | |
| Welke figuur geeft het beste het beloop van de dosering van | | | | | | | - geen verhoging | | | | | | | | | | | | | | - geleidelijke | | | | | - sterke verhoging | | | | | |
| de morfine en/of een ander opiaat weer in de laatste 3 dagen | | | | | | |  | | | | | | | | | | | | | | verhoging | | | | | laatste dag | | | | | |
| vóór het overlijden van de patiënt? | | | | | | | 3 2 1 0 | | | | | | | | | | | | | | 3 2 1 0 | | | | | 3 2 1 0 | | | | | |
|  | | | | | | |  | | | | | | | | | | | | | | | | | | | | | | | | |

|  | | | | | | | | | |  | | | | | | | | | | | | |
| --- | --- | --- | --- | --- | --- | --- | --- | --- | --- | --- | --- | --- | --- | --- | --- | --- | --- | --- | --- | --- | --- | --- |
| 1. Heeft de patiënt benzodiazepine(s) toegediend gekregen | | | | | | | | | |  | | | ja | | | | | | | | | |
| tijdens de laatste 24 uur vóór het overlijden? | | | | | | | | | |  | | | neen 🡺 **door naar vraag 27** | | | | | | | | | |
|  | | | | | | | | | |  | | | | | | | | | | | | |
|  | | | | | | | | | |  | *Naam* | | | | | | | | *Toedieningswijze* | *Dosering*  *(laatste 24 uur)* | | |
| Naam van het (de) middel(en), toedieningswijze en dosering | | | | | | | | | |  |  | | | | | | | |  |  |  | |
| in de laatste 24 uur vóór het overlijden? | | | | | | | | | |  |  | | | | | | | |  |  |  | |
|  | | | | | | | | | |  |  | | | | | | | |  |  |  | |
|  | | | | | | | | | |  | | | | | | | | | | | | |
|  | | | | | | | | |  | | | | | | | | | | | | | |
| 1. Heeft de patiënt een uitdrukkelijk verzoek om | | | | | | | | |  | | | ja | | | | | | | | | | |
| levensbeëindiging gedaan dat niet werd ingewilligd? | | | | | | | | |  | | | neen 🡺 **door naar vraag 28** | | | | | | | | | | |
|  | | | | | | | | |  | | | | | | | | | | | | | |
| Om welke reden(en) werd dit verzoek niet ingewilligd? | | | | | | | | |  | | | de patiënt overleed voordat het tot inwilliging kon komen | | | | | | | | | | |
| *– meerdere antwoorden mogelijk –* | | | | | | | | |  | | | de patiënt was niet terminaal ziek | | | | | | | | | | |
|  | | | | | | | | |  | | | het lijden was niet ondraaglijk | | | | | | | | | | |
|  | | | | | | | | |  | | | de medische toestand was niet uitzichtloos | | | | | | | | | | |
|  | | | | | | | | |  | | | het was geen weloverwogen verzoek | | | | | | | | | | |
|  | | | | | | | | |  | | | het was geen vrijwillig verzoek | | | | | | | | | | |
|  | | | | | | | | |  | | | de patiënt trok het verzoek weer in | | | | | | | | | | |
|  | | | | | | | | |  | | | vanwege instellingsbeleid | | | | | | | | | | |
|  | | | | | | | | |  | | | vanwege principiële bezwaren tegen levensbeëindiging | | | | | | | | | | |
|  | | | | | | | | |  | | | uit vrees voor juridische consequenties | | | | | | | | | | |
|  | | | | | | | | |  | | | andere reden, desgewenst toelichten bij vraag 29 | | | | | | | | | | |
|  | | | | | | | | |  | | | | | | | | | | | | | |
| 1. Hoe tevreden bent u, achteraf bekeken, met het verloop van het levenseinde van de patiënt? | | | | | | | | | | | | | | | | | | | | | | |
|  | | | | | | | | | | | | | | | | | | | | | | |
|  | | 0 | 1 | 2 | 3 | 4 | 5 | 6 | | | | | | 7 | 8 | 9 | 10 |  | | | | |
| niet tevreden | |  |  |  |  |  |  |  | | | | | |  |  |  |  | heel tevreden | | | | |
|  | |  | | | | | | | | | | | | | | | |  | | | | |
|  | |  | | | | | | | | | | | | | | | |  | | | | |
| Hoe schat u de tevredenheid van de naasten hieromtrent in? | | | | | | | | | | | | | | | | | | | | | | |
|  | | | | | | | | | | | | | | | | | | | | | | |
|  | | 0 | 1 | 2 | 3 | 4 | 5 | 6 | | | | | | 7 | 8 | 9 | 10 |  | | | | |
| niet tevreden | |  |  |  |  |  |  |  | | | | | |  |  |  |  | heel tevreden | | | | |
|  | |  | | | | | | | | | | | | | | | |  | | | | |
|  | | | | | | | | | | | | | | | | | | | | | | |
| ***Toelichting*** | | | | | | | | | | | | | | | | | | | | | | |
|  | | | | | | | | | | | | | | | | | | | | | | |
| 1. Als bepaalde van uw antwoorden volgens u nog verdere verduidelijking behoeven, kunt u dit hier neerschrijven. | | | | | | | | | | | | | | | | | | | | | | |
|  | | | | | | | | | | | | | | | | | | | | | | |
|  | | | | | | | | | | | | | | | | | | | | | | |
|  |  | | | | | | | | | | | | | | | | | | | | |  |
|  |  | | | | | | | | | | | | | | | | | | | | |  |
|  |  | | | | | | | | | | | | | | | | | | | | |  |
|  |  | | | | | | | | | | | | | | | | | | | | |  |
|  |  | | | | | | | | | | | | | | | | | | | | |  |
|  |  | | | | | | | | | | | | | | | | | | | | |  |
|  |  | | | | | | | | | | | | | | | | | | | | |  |
|  |  | | | | | | | | | | | | | | | | | | | | |  |
|  |  | | | | | | | | | | | | | | | | | | | | |  |
|  |  | | | | | | | | | | | | | | | | | | | | |  |
|  |  | | | | | | | | | | | | | | | | | | | | |  |
|  |  | | | | | | | | | | | | | | | | | | | | |  |
|  |  | | | | | | | | | | | | | | | | | | | | |  |
|  | | | | | | | | | | | | | | | | | | | | | | |

**HARTELIJK DANK VOOR UW DEELNAME !**
